# Application of 3D Scanning Method to Assess Mounting Holes’ Shape Instability of Pinewood

**DOI:** 10.3390/ma16052053

**Published:** 2023-03-02

**Authors:** Maciej Sydor, Jerzy Majka, Michał Rychlik, Wojciech Turbański

**Affiliations:** 1 Department of Woodworking and Fundamentals of Machine Design, Faculty of Forestry and Wood Technology, Poznań University of Life Sciences, 60-637 Poznań, Poland; 2 Euroline sp. z o.o., 64-100 Leszno, Poland; 3 Department of Engineering Mechanics and Thermal Techniques, Faculty of Forestry and Wood Technology, Poznań University of Life Sciences, 60-637 Poznań, Poland; 4 Institute of Applied Mechanics, Faculty of Mechanical Engineering, Poznan University of Technology, 60-965 Poznań, Poland

**Keywords:** Scots pine, drilling, swelling, shrinkage anisotropy, effective diameter, plug gauge method, synthetic gypsum cast, optical laser scanner, 3D scanning, furniture assembly

## Abstract

Swelling and shrinkage anisotropy affect the susceptibility to an assembly of wooden elements by changing designed clearances or interference fits. This work described the new method to measure mounting holes’ moisture-induced shape instability and its verification using three sets of twin samples made of Scots pinewood. Each set of samples contained a pair with different grain patterns. All samples were conditioned under reference conditions (relative air humidity–RH = 60% and temperature 20 °C), and their moisture content (MC) reached equilibrium (10.7 ± 0.1%). On the side of each sample, the seven mounting holes of 12 mm in diameter were drilled. Immediately after drilling, Set 1 was used to measure the effective hole diameter with 15 cylindrical plug-gauges with diameters of 0.05 mm step, while Set 2 and Set 3 were separately re-seasoned by six months in two extreme conditions. Set 2 was conditioned with air at 85% RH (reached an equilibrium MC of 16.6 ± 0.5%), while Set 3 was exposed to air at 35% RH (reached an equilibrium MC of 7.6 ± 0.1%). Results of the plug gauge tests highlighted that holes in the samples subjected to swelling (Set 2) increased an effective diameter in the range of 12.2–12.3 mm (1.7–2.5%), while samples subjected to shrinking (Set 3) reduced the effective diameter to 11.9–11.95 mm (0.8–0.4%). To accurately reproduce the complex shape of the deformation, gypsum casts of holes were made. The 3D optical scanning method was used to read the gypsum casts’ shape and dimensions. The 3D surface map of deviations analysis provided more detailed information than the plug-gauge test results. Both the shrinking and swelling of the samples changed the shapes and sizes of the holes, but shrinking reduced the effective diameter of the hole more than swelling increased it. The moisture-induced changes in the shape of holes are complex: the holes ovalized with a different range, depending on the wood grain pattern and hole depth, and were slightly extended in diameter at the bottom. Our study provides a new way to measure 3D hole initial shape changes in wooden elements during desorption and absorption.

## 1. Introduction

Most properties of wood are dependent on its moisture content (MC). This relationship occurs below the fiber saturation point (FSP). Tiemann [1] first defined FSP as the moisture content of the wood in which the cell walls are thoroughly saturated with bound water, but there is no free water in the lumens. In further studies, Tiemann examined the effect of MC on the strength and stiffness of wood, and the FSP was redefined as the MC corresponding to abrupt changes in the physical and mechanical properties of wood [2]. There is a variation in FSP values between wood species of 16% for western redcedar (*Thuja plicata*) [3] to 32% for *Tilia Americana* [2].

Furthermore, the actual FSP value depends on the temperature of the wood and decreases by approximately 0.1% per 1 °C. Some sources suggest that hardwoods may have a slightly higher FSP than softwoods because hardwood cells are more densely packed and have thicker cell walls [4]. Generally, the FSP range of 25–30% at 20 °C can be assumed to be a reasonable estimate for most temperate wood species. MC dimensional changes below the FSP, including shrinkage or swelling, appear most significant in wood science and engineering [2].

Many scientific papers reviewed the factors determining wood shrinking and swelling, including the influence of MC, temperature, and relative humidity (RH), and discussed how dimensional wood changes can be appropriately measured and predicted [5]. A characteristic feature of wood shrinkage is its anisotropy, which is dependent on the anatomical direction. The average shrinkage value in the longitudinal (L) direction is less than 1% [5]. From a practical point of view, the dimensional MC changes of wood occur in the tangential (T) and radial (R) anatomical direction [6,7,8,9]. The shrinkage or swelling in the tangential direction can range from more than one to nearly three times more than in the radial direction. The T/R shrinkage anisotropy is explained by the “ray restraint” theory [10], the early-wood–late-wood interaction theory [11], the differences in the strain of the cell wall layers theory, and the angles of the longitudinal fibril arrangement within the S_2_ layer theory [1]. The wood shrinkage also depends on the drying method used. Previous studies reported that shrinkage from the green to an oven-dry state of *Pinus koraiensis* in the tangential and radial direction ranged from 6.5 to 7.4% and from 2.5 to 2.8%, respectively [12].

The engineering tolerances for holes drilled into solid wood elements are specified in terms of the nominal diameter and location. Engineering tolerances are a rationalization of the inaccuracy of woodworking. The deviation of nominal diameter and the shape distortion of the drilled holes are cumulative of manufacturing variables: cutting speed, actual sharpness of the tool, machine spindle rigidity, clamping rigidity, and thermal expansion. Therefore, the drilling is not correct, and the dimensions and shape of the holes are inaccurate. Commercial twist drills may have a diameter slightly smaller than the nominal; however, the actual hole diameter will likely be larger than the nominal diameter of the used tool. The inaccuracy of wood processing both the MC changes in the dimensions of wooden elements caused by changes in air parameters makes the shapes of the holes far from cylindrical. In the previous study [13], we measured commonly used cylindrical plug go/no-go gauges changes in the effective diameter of drilled holes at the edge and the face of Scots pine plywood blanks under conditions of exposure to air with different RH. It was found that the change in effective diameter after air conditioning depended on the grain pattern of the blank and the character of changes in MC of the wood. Changes in MC caused by a decrease in RH from 60 to 35% (causing shrinkage) significantly reduced the effective diameter of the hole.

In comparison, changes in MC caused by an increase in RH from 60 to 85% (causing swelling) only slightly increased the effective diameter of the hole. Moreover, it was observed that changing the shape of the wood MC distorted the hole from round to oval. Significant changes in the shape of the drilled holes were observed in the case of timber blanks with a tangential grain pattern. Therefore, the grain pattern is critical to designing an engineering fit in solid wood elements for assembly in variable ambient air conditions.

**Figure 1:**
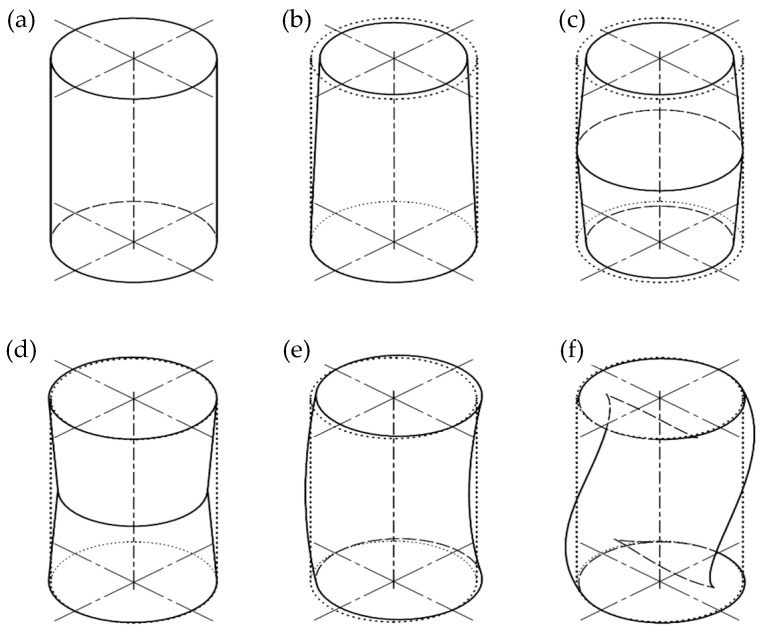
Examples of cylindricity deformations: (**a**)—ideal cylinder, (**b**)—conicity deviation, (**c**)—barrel-like deviation, (**d**)—saddle-like deviation, (**e**)—median line deviation, (**f**)—complex shape deviation (source [13]).

As a result of changes in MC, wood swells or shrinks. Then, cylindrical holes drilled in the wood elements may become deformed—various factors, such as the natural cylindrical ortotrophy and irregularity of wood structure, affect these shape changes. Figure 1 provides an example of how these changes can affect the shape of the holes.

Changing the MC of the wooden elements affects many functional properties of the wooden products, e.g., changing the load capacity [14]. The assembly of wooden furniture elements with tangential or radial grain patterns can be difficult or even impossible because the shape of the holes in the wood may become deformed due to many cycles of swelling and shrinkage occurring with different intensities. The commonly used cylindrical plug go/no-go gauges measuring method allowed only to confirm the compliance of the physical dimension with the dimension via a go/no-go test. Such a test does not give complete knowledge of the shape of the hole. The shape of the drilled holes may deviate from the cylindrical pattern due to inaccuracies in the drilling process and the described dimensional variation caused by shrinking/swelling anisotropy of the wood due to changes in its MC. This article aimed to propose a more appropriate method to measure the instability of the shape of holes drilled in solid wooden elements.

## 2. Materials and Methods

### 2.1. The Test Sample Preparation

**Figure 2:**
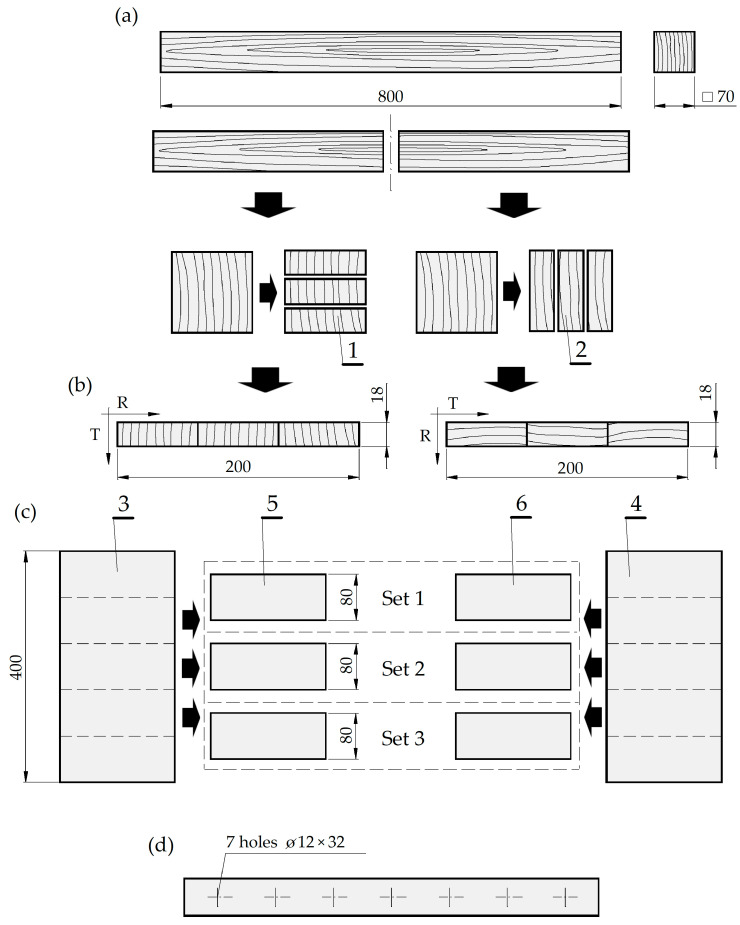
Scheme of samples preparation: (**a**)—initial cutting, (**b**)—assembling and gluing radial (R) and tangential (T) grain pattern blank, (**c**)—final cutting of samples sets, (**d**)—the holes drilling pattern in the narrow side of the sample, (1, 2—radial and tangential pieces of blank, respectively, 3, 4—radial and tangential grain pattern blank, respectively, 5, 6—final radial and tangential twin samples, respectively, T, R—tangential and radial anatomical direction, respectively; the dimension in mm).

The test samples were prepared using kiln-dried Scots pine (*Pinus sylvestris* L.) flat-sawn timber with a target moisture content (MC) of 12 ± 2%. The sample preparation procedure included: selecting clear sapwood planks without visible defects, gluing and cutting the planks, seasoning, and finally, drilling holes (Figure 2). The two parts of the selected plank were cut separately to tangential (T) and radial (R) direction (Figure 2a). Then, the planks were assembled and glued to the plank with radial and tangential grain patterns (Figure 2b). Finally, three sets of samples were produced (Figure 2c). Each set consisted of two (twin) samples, one with tangential grain pattern and second with radial grain pattern. The final dimensions of the prepared samples were 18 mm × 200 mm × 80 mm in thickness, width, and length, respectively.

In the first phase of experiments, all test samples were initially air-conditioned for four months over sodium bromide (NaBr) in a container with forced air circulation under reference conditions, i.e., RH = 60 ± 1% and *t* = 20 ± 1 °C, to obtain a constant equilibrium mass. After air conditioning, the MC reached 10.7 ± 0.1%. In the next phase, 7 holes were drilled at all samples’ edges (on the narrow side). Figure 2d presents the drilling pattern (diameter 12 mm, depth 32 mm, spacing 32 mm—the standard of the “system 32” in the furniture industry). The holes were drilled with new, fully sharpened carbide blade blind bits with a nominal diameter of 12 mm and a length of 77 mm (HW/D12/NL45/S10 × 30/GL77/RE type, Leitz GmbH & Co. KG, Oberkochen, Germany). An industrial CNC machine tool (Homag Centateq P-110, Schopfloch, Germany) was used for drilling (parameters of drilling: rotation speed 8000 rpm, cutting speed *v_c_* = 300 m/min, feed rate 7.2 m/min, which means a chip load *f_z_* = 0.45 mm/tooth).

Immediately after drilling, Set 1 was used to measure the holes’ effective diameter with plug gauges, while Set 2 and Set 3 were separately placed in other containers in two extreme conditions that differed in air RH at 20°C from the reference conditions by +25% (Set 2) and by −25% (Set 3). It was assumed that these conditions corresponded to practical differences in humidity in which wooden elements could be stored before assembly. Set 2 was exposed to a higher RH of 85%, causing swelling. In contrast, Set 3 was exposed to a lower RH of 35%, causing shrinking. Potassium chloride (KCl), sodium bromide (NaBr), and calcium chloride (CaCl_2_·6H_2_O) were used to obtain conditions corresponding to the RH of 85, 60, and 35%, respectively. These salt solutions were chosen to obtain constant air RH during the air conditioning of test samples. Sets 2 and 3 were conditioned until they reached a constant mass, which took six months. Immediately after conditioning, Sets 2 and 3 were used to measure effective diameter with plug gauges.

### 2.2. The Effective Diameter Measurement

The effective diameter of the drilled hole was assumed to be the maximum diameter of a cylinder that could be inserted manually into the hole. A five-stage dimensionless plug-gauge fitting scale (Table 1) was used to recognize all intermediate diameters between the lower and upper limit gauges. The scale was adopted from previous research [13]. This experiment used a set of 15 cylindrical plug gauges with diameters ranging from 11.60 to 12.40 mm (every 0.05 mm). During the measurement, the plug gauges were successively manually inserted into the drilled holes in diameter order. On this basis, the limit diameters were determined. Above the lower limit diameter, there was resistance, while above the upper diameter, it was impossible to insert the gauge into the drilled hole. The effective diameter was determined as the modal value of 7 measurements.

### 2.3. The Moisture Content Measurement

The MC of examined samples was measured according to the oven dry method (EN 13183-1 2002 [15]) and calculated using the following formula:

$$MC=\frac{m_{1}-m_{0}}{m_{0}}\cdot 100\%$$

where *m*_1_ was the mass of wood with moisture content and *m*_0_ was the oven-dry mass of wood.

An electronic laboratory balance with measurement inaccuracy Δ*m* = ± 0.001 g (type PA 213/1, OHAUS, Parsippany, NJ, USA) was used to measure the mass of the wood samples. The MC was calculated for each air RH condition (i.e., 35, 60, and 85%) as an average of two calculations. The MC of all samples, initially conditioned in the air with RH = 60% (Set 1), equilibrated at 10.7 ± 0.1%. Exposure to air with RH = 85% (Set 2) increased the MC of the wood by 5.9% (to 16.6 ± 0.5%); exposure to RH = 35% (Set 3) decreased the MC by 2.9% (to 7.6 ± 0.1%).

### 2.4. The 3D Scanning of Hole Shapes

**Figure 3:**
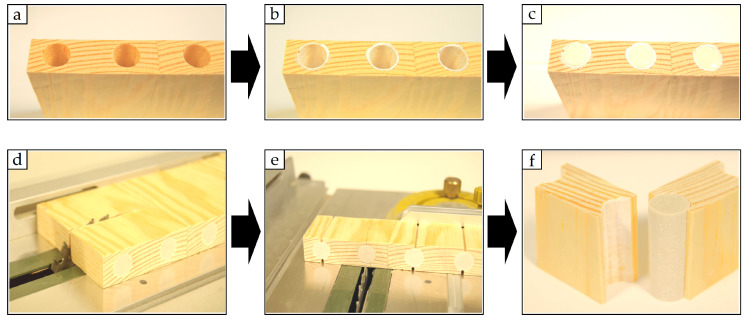
Scheme of preparation and extraction of the hole counter-samples (**a**—wood sample with drilled holes, **b**—wood sample with applied coating, **c**—wood sample immediately after filling the holes, **d**,**e**—cutting of wood samples with hardened casts, **f**—the final shape of extracted counter-sample).

The shape of the drilled holes could not be optically 3D-scanned directly, so it was decided to produce a gypsum cast. Synthetic casting gypsum (POLYCORE, Świdnik, Poland) with less than 0.15% linear expansion was used to produce the casts. The gypsum casts were the counter-samples of drilled holes. The counter-sample preparation is shown in Figure 3. The surface of all drilled holes was coated with silicone grease (AG TermoPasty, Sokoły, Poland) to protect against water penetration from the gypsum-water mixture (Figure 3b). Immediately after coating, the drilled holes were filled with a gypsum-water mixture (Figure 3c). The curing time was approx. 10 min. The hardened counter-samples were carefully extracted using the table saw FET (PROXXON MIKROMOT System, Föhren, Germany) (Figure 3d–f).

The gypsum counter-samples were scanned using a 3D optical laser scanner (model LPX-600, Roland DG, Hamamatsu, Japan) with a noncontact laser sensor (wavelength ranged from 645 to 660 nm; maximum output up to 0.39 μW). Spot beam triangulation and the scanning pitch for rotary scanning with parameters: circumference from 0.18° to 3.6°, height direction from 0.2 to 406.4 mm, and repeat accuracy of ±0.05 mm were used. Each of the measured counter-samples was placed in a vertical position on the rotary measuring table of the 3D scanner. The vertical axis of the counter-sample coincided with the axis of rotation of the measuring table. A trial rotation was performed to minimize the deviation of the counter-sample vertical axis to the axis of the measuring table. If necessary, the position of the counter-sample was corrected. Then, an automated 3D scanning of the examined surface of the samples was performed with the following measurement settings: circumferential pitch of 0.18°, height direction pitch: of 0.2 mm, and rotation of the measuring table at a constant speed.

**Figure 4:**
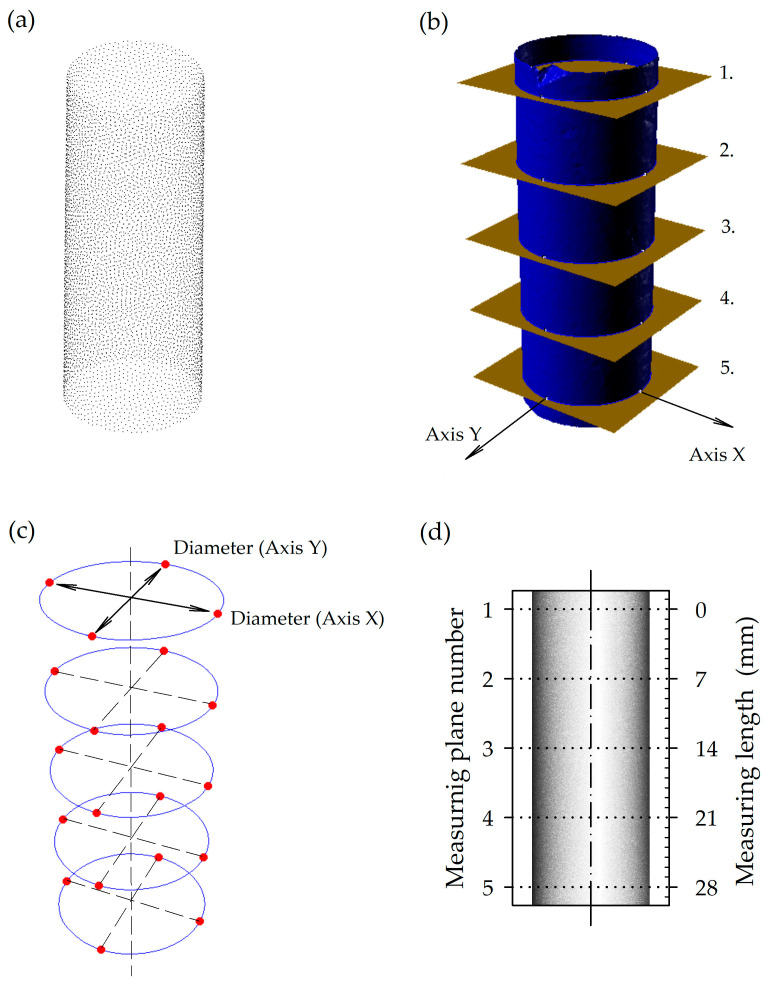
Processing of 3D scanning results: (**a**)—point cloud, (**b**)—surface model based on the point cloud with five cross sections, (**c**)—cross-section figures with measure points, (**d**)—front view of gypsum counter-sample with spaced measuring planes.

As a result of the 3D scanning of each examined counter-sample with a nominal diameter of 12 mm and a length of 32 mm, a cloud containing approximately 320,000 measurement points was obtained; on average, the horizontal distance between the measurement points was 0.018 mm. The measurement time for a single sample was approx. 21 min. The procedure for processing the 3D scan results is presented in Figure 4.

The point cloud of each examined counter-sample was exported to Geomagic software (3D Systems, Inc., Rock Hill, SC, USA), and a 3D surface model was generated using a triangle mesh method. Next, each scanned model was registered, which spatially oriented the model of the counter-sample to the reference sample, with ideal dimensions and orientation in Cartesian coordinates (XYZ). In the next step, the 3D-scanned models were subjected to measuring diameters on five control planes (Rhinoceros software, Robert McNeel & Associates, Seattle, WA, USA). The central control plane was 16 mm from the lower base of the counter-sample, and the other planes were symmetrically 7 mm spaced. The upper and lower measuring planes, i.e., 1 and 5, corresponded to the inlet and the bottom of the drilled hole, respectively. In each control plane, measurements were carried out in two perpendicular directions (X, Y), corresponding to the radial (R) and tangential (T) anatomical direction.

## 3. Results

### 3.1. The Results of the Effective Diameter Measurement

**Figure 5:**
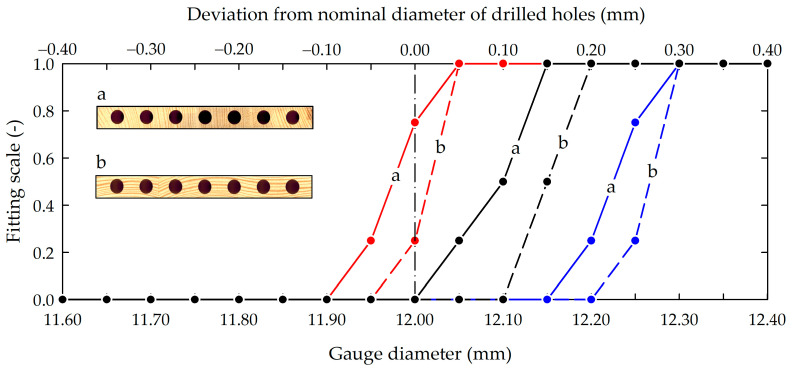
Results of the effective diameter measurements by plug gauge method according to grain pattern and moisture content of Scot’s pine planks (a, b—radial and tangential grain pattern, respectively; black dots and lines—conditions corresponding to RH = 60% (reference); blue dots and lines—conditions causing swelling of samples (increased air RH to 85%); red dots and lines—conditions causing shrinking of samples (decreased air RH to 35%); dots represents modal values (*n* = 7); dash-dot line–nominal diameter of drilled holes (*d* = 12 mm); limit values of fitting scale: 0—loose running (larger clearance, can be assembled), 1—interference fits (press fits, cannot be assembled).

Figure 5 shows the results of measuring the effective diameter of the drilled holes using the plug gauges. Table 1 shows the plug-gauge fitting scale used.

Figure 5 shows that in the case of samples seasoned with reference conditions (air RH = 60%), the upper limit diameter of the gauge that could be manually inserted into the hole was 12.0–12.1 mm (this was a diameter equal to or greater than 0.1 mm of the nominal diameter of the hole). A decrease in moisture content (MC) (change RH from 60 to 35%) caused the shrinking of the samples, resulting in an effective diameter in the range of 11.9–11.95 mm, while an increase in the MC (change RH from 60 to 85%) caused swelling of the samples, and plugging resistance was observed for gauges with a diameter of 12.2–12.3 mm. This means that the shrinking of wood samples reduced the effective diameter of the drilled holes, while the swelling of samples increased the effective diameter. In the case of radial grain pattern planks (sample “a” in Figure 5), the upper-limiting diameter of the gauge that could be freely inserted into the hole was smaller than in the case of tangential grain pattern planks (sample “b” in Figure 5). It highlighted that the changes in the dimensions of holes depended on grain pattern and the direction of changes in MC (adsorption/desorption).

### 3.2. The Results of 3D Scanning of Hole Shapes

**Figure 6:**
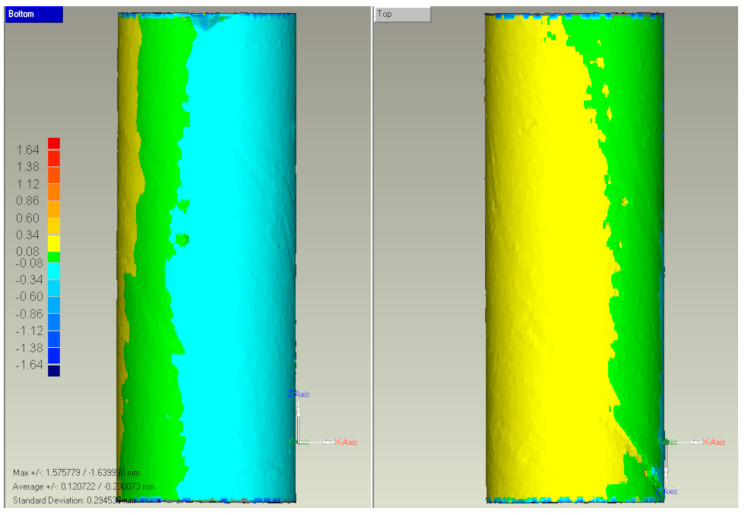
Example 3D surface deviations map of examined gypsum counter-sample from the cylindrical shape.

The results of 3D-scanning the gypsum counter-samples were virtual models capable of comparing with a theoretical cylinder with a diameter of 12 mm and a length of 32 mm. Figure 6 shows an example of the 3D scan result.

**Figure 7:**
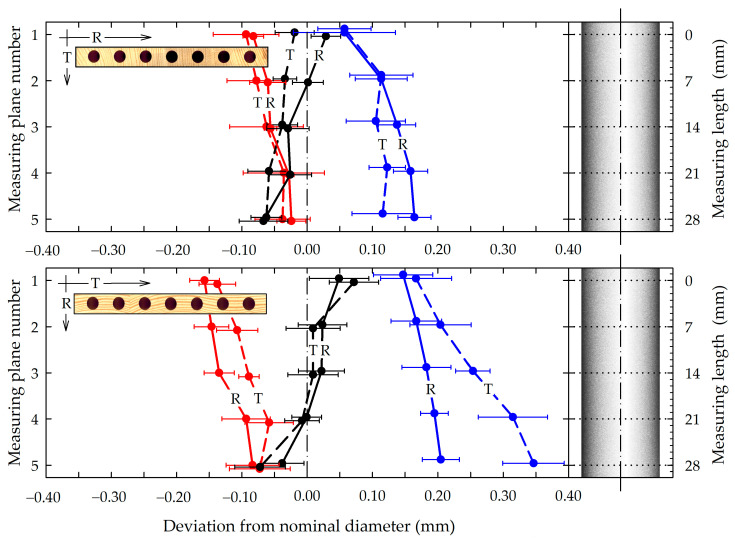
Deviation from a nominal diameter of drilled holes according to grain pattern and MC of radial (**above**) and tangential (**bottom**) samples; notations: black lines–conditions corresponding to RH = 60% (reference); blue lines—conditions causing swelling of samples (increased air RH to 85%); red lines—conditions causing shrinking of samples (decreased air RH to 35%); dots represent average ± standard deviation (*n* = 7); R—radial anatomical direction; T—tangential anatomical direction.

As mentioned, six series of seven counter-samples of gypsum were analyzed, two for radial and tangential grain pattern planks for three seasoning conditions (RH = 60, 85, and 35%). The 3D scans of each series were averaged, thus obtaining six averaged 3D scans of gypsum counter-samples. A CAD model of an ideal cylinder with a diameter of 12 mm and height of 32 mm was superimposed on these averaged 3D scans. The result of this imposition is shown in Figure 7. These 3D scan results indicated that the hole counter-samples had shapes that deviated from the ideal cylinder. It was almost a cylinder but with ellipsoidal cross-sections. In the figure, the black color indicates changes in the dimensions of reference samples (Set 1); the blue color indicates the dimensional changes in the diameter of the holes in the samples exposed to high air RH (Set 2), and the red color showed the dimensional changes in the samples exposed to low air RH (Set 3). The distance between pairs of lines of the same color showed the ovality of the holes. The ovalization was greater in Set 2. It also seemed more intense in the bottom of the holes (measuring planes 4 and 5). The shapes of the cross-sections depended on the distance from the drilled hole’s inlet. In Figure 7, it can also be observed that the holes were slightly tapered. The slope of the black lines to the right indicates that the diameter of the holes in Set 1 increased with the hole depth (the holes had a smaller diameter in measurement plane 1 than in measurement plane 5). The opposite phenomenon was observed for the holes of Sets 2 and 3. The line deviation from the vertical indicates the rate of increase or decrease in diameter. The distances between the pairs of lines in different colors showed the scale of dimensional changes in the holes’ diameters caused by exposure to high-air RH and low-air RH.

The 3D scanning of the hole counter-samples revealed that reducing the MC of wood samples reduced the effective diameter of the holes (Figure 7), and increasing the MC increased it. These results were consistent with the plug-gauge measurement results shown in Figure 5.

Furthermore, Figure 7 shows that the changes in the effective diameters of the holes drilled on the side of planks with radial grain patterns were considerably smaller than those with tangential grain patterns subjected to identical MC changes. The most significant deformation (dimensional and shape changes) in the hole axis was confirmed in the case of tangential plank subjected to swelling. The scope and nature of these deformations depended on the direction of changes in wood moisture. The analysis of the diameter deviation from the nominal value indicates that changes/deformations of shapes caused by changes in the MC of the wood were unequal. This fact indicates that deformation of the shape of holes drilled in the side surfaces of solid wood elements under the influence of changes in its humidity should not be considered only as an effect of wood shrinking and swelling anisotropy–and, therefore, only in the plane perpendicular to the axis of the hole–but should also be considered in the hole depth.

## 4. Discussion

Wood constantly exchanges moisture with the ambient air, tending to an equilibrium moisture content (MC). The wood used under real-life conditions usually never attains equilibrium MC due to the long adsorption–desorption phenomenon and the varying nature of the ambient conditions, such as air RH and temperature [16]. The slope of wood MC changes during adsorption is different than in desorption. The difference in the relationship between MC and RH during adsorption and desorption is known as sorption hysteresis [17]. This causes constant dimensional changes in the wooden elements [14]. The direction and the dimensional range of the changes depend on the direction of the sorption phenomena, i.e., adsorption and desorption [18]. Our research results confirmed these observations. These moisture-induced dimension changes in pine samples caused changes in the dimensions of holes made in them. The measured swelling and shrinking were not symmetrical. Long-term exposure to air with higher RH (of 85%) resulted in an increase of the tested samples’ MC by 5.9% (from 10.7 ± 0.1% to 16.6 ± 0.5%) while the exposure to air with lower RH (of 35%) decreased the samples’ MC by 2.9% (to MC = 7.6 ± 0.1%). Change in air RH by +25% and by −25% causes asymmetric changes in samples’ MC. The change in wood MC caused by increased air RH by 25% was approximately twice more than the change in wood MC caused by air with a decreased RH in the same range. A similar effect was reported in previous studies [13]. The explanation of this phenomenon is the sorption hysteresis mentioned above. During adsorption, wood can increase MC rapidly at a low air RH. The MC may not decrease as rapidly during desorption when the air RH decreases, as some MC may remain in bound form and only be released at very low RH. This leads to a difference in the relationship between MC and RH during adsorption and desorption [8,19].

The results indicate that the drilled holes, although made with a new high-end tool and a new industrial CNC machine, were not ideally cylindrical. These holes had slightly smaller diameters in their lower part. This phenomenon was visible in 3D scanning of the counter-samples (gypsum casts of the holes). Therefore, the hole diameter inaccuracy was not caused by dulling of the tool [20]. It should be mentioned that the diameter ratio to the length of the drilling was large (the diameter of the drill bit was 12 mm, and the depth of the drilling was 40 mm); therefore, the drill [21,22] should be excluded too. A potential cause of taper-shaped holes can be the influence of chips that widen the hole at the bottom.

Under typical industrial conditions, wooden elements are exposed to varying ambient RH during transport or storage before being built into the produced furniture [23]. The effective diameter of the hole, i.e., the diameter of the cylinder gauge that can be freely inserted into such a hole, determines the susceptibility to assembly. The undesirable conversion of designed clearance into an interference fit during robotic assembly can make it difficult or even impossible to assemble the furniture elements by robots because industrial robots are usually inefficient at measuring and regulating forces required during assembly [24]. Therefore, the robot is more susceptible to changing the parameters of the assembled elements than a human [25]. Maintaining predictable effective hole diameters is particularly important when automating the assembly process because the pin insertion task is a benchmark task in robotic furniture assembly and is a basis for evaluating the success rate [26].

Changes in the shapes of drilled holes under the influence of changes in wood MC occur with different ranges and scales depending on the anatomical direction [13,27], but they also occur with different ranges at a depth of the hole. With water absorption, the hole in its upper measuring plane 1 (shown in Figure 4 and Figure 7) increased in diameter more, indicating that absorption was greater there than in the deepest measuring plane of the hole (no. 5). However, the effective diameter reduction in the same upper measuring planes was less during adsorption than in its deeper measuring planes. In other words, increasing the MC of a sample affected the upper part of the hole, while decreased MC deformed the deepest part of the hole.

## 5. Conclusions

The shrinkage and swelling anisotropy affect the susceptibility to the mounting of elements of wooden furniture. The work presented a new method for accurate measurement of the shape of holes. The described experimental study identified the nature and value of the hole deformation and confirmed the usefulness of the 3D scanning technique used to measure the phenomena studied. The experiment included inducing swelling and shrinkage of pinewood elements with bored holes. Changes in moisture content (MC) were caused by a long-term increase and a decrease in RH of 25%. Effective hole diameters were measured using plug gauges, while the deformed shape of the holes was “read” with a 3D scanner. Based on the analysis of the results, the following general conclusions can be drawn:

1. The exposure to air with a decreased RH of 60% to 35% decreased the equilibrium MC of the test samples by 3%. In contrast, an increase in the RH to 85% increased the equilibrium MC by 5.9%. The tested pinewood adsorbed water to times more then it desorbed. This was due to sorption hysteresis.
2. Shapes of drilled holes were not ideally cylindrical. These holes were slightly wider at their bottoms. A potential cause of the increase in diameter with the hole depth may have been the cutting action of the chips generated during drilling. The deeper the hole, the more chips were not evacuated, and these chips could increase the hole diameter when drilling.
3. Susceptibility to the assembly of wood elements depended on the direction of changes in the MC of wood. The desorption (a decrease in MC) negatively affected this susceptibility, significantly reducing the effective diameter of the holes; the cross-sections of holes deformed into ellipses, thus significantly reducing the diameter of the cylindrical gauge that could be inserted manually into such a deformed hole. The presented results and analyses are potentially helpful in optimizing the production of solid wood elements for robotic assembly.
4. Changes in the shapes of holes under the influence of changes in wood MC are complex. The different intensity of the hole diameter changes concerning the depth of the hole were probably caused by different rates of water absorption and desorption occurring at a depth of the hole. The wood in the upper parts of the hole absorbed water more quickly and swelled more than the bottom. The nature of shrinkage and swelling and the range of holes’ deformations depended on the direction of the air’s impact on the wood (i.e., increase or decrease in air RH) and the grain pattern in the element in which the holes were drilled.
5. 3D scanning of gypsum cast of holes provides very detailed information about the shape of the holes. The study confirmed the usefulness of the 3D scanning technique to assess the dimensional and shape instability of mounting holes in solid wood. The analysis of 3D scanning results confirmed the assumption that the classic “plug-gauging” technique is insufficient to properly assess the dimensional and shape instability of holes in solid wood affected by its MC changes. The study provides new insights into measuring 3D hole shape changes in wooden elements using 3D optical scanning.

## Figures and Tables

**Table 1:** Values and Description of the Assumed Plug-Gauge Fitting Scale (according to [13]).

Value	Description
0.00	Loose running (larger clearance, can be assembled)
0.25	Close running (small clearance, can be assembled)
0.50	Location (very close clearance, can be assembled)
0.75	Transition fits (negligible clearance, can be assembled)
1.00	Interference fits (press fits, cannot be assembled)

## Data Availability

All data generated during the study appear in the submitted article.
